# Rosiglitazone-induced changes in the oxidative stress metabolism and fatty acid composition in relation with trace element status in the primary adipocytes

**DOI:** 10.2478/jomb-2019-0041

**Published:** 2020-09-02

**Authors:** Duygu Aydemir, Ehsan Sarayloo, Ulusu Nuriye Nuray

**Affiliations:** 1 Koç University, School of Medicine, Rumelifeneri Yolu, Sariyer, Istanbul, Turkey

**Keywords:** rosiglitazone, oxidative stress, trace elements, minerals, fatty acid, rosiglitazon, oksidativni stres, elementi u tragovima, minerali, masna kiselina

## Abstract

**Background:**

Metabolic syndrome, obesity and type 2 diabetes are metabolic disorders characterized by the insulin resistance and the impairment in the insulin secretion. Since impairment in the oxidative stress and adipocyte metabolism contribute to the formation of obesity and diabetes, targeting adipose tissue can be considered as an effective approach to fight against them. Rosiglitazone is used for treatment for patients with type 2 diabetes via inducing lipogenesis and transdifferentiation of white adipose tissue into brown adipose tissue. Since the development of such therapeutics is required to control the formation and function of brown fat cells, we aimed to reveal possible molecular mechanisms behind rosiglitazone induced biochemical changes in the adipose tissue.

**Methods:**

Cells were expanded in the adipocyte culture medium supplemented with 5 µg/mL insulin following 2 days' induction. After those cells were treated with rosiglitazone 0, 0.13 mol/L and 10 µmol/L rosiglitazone for 48 hours and at 8th day, cells were collected and stored at -80 °C. Then the cells were used to evaluate antioxidant enzyme activities, mineral and trace element levels and fatty acid composition.

**Results:**

Glucose-6-phosphate dehydrogenase and glutathione reductase significantly reduced in rosiglitazone-treated groups compared to the control. Na, Mg, K, Ca, Cr, Fe, Ni, Cu, Zn, Rb, Sr, Cs, Ba and Pb were determined in the cell lysates via ICP-MS. Also, relative FAME content decreased in the rosiglitazone-treated groups compared to the control.

**Conclusions:**

Rosiglitazone treatment at low doses showed promising results which may promote brown adipose tissue formation.

## Abstract (sr)

**Uvod:**

Metabolički sindrom, gojaznost i dijabetes tip 2 su metabolički poremećaji za koje je karakteristična otpornost na insulin i poremećaj lučenja insulina. S obzirom da poremećaj oksidativnog stresa i metabolizma adipocita doprinosi gojaznosti i dijabetesa, ciljanje masnog tkiva se može smatrati efikasnim pristupom u borbi protiv njih. Rosiglitazon se koristi za lečenje pacijenata sa dijabetesom tipa 2 putem indukcije lipogeneze i transdiferencijacije belog masnog tkiva u mrko masno tkivo. Pošto je razvoj takvih terapija potreban za kontrolu stvaranja i funkcije mrkih masnih ćelija, želeli smo da otkrijemo moguće molekularne mehanizme koji stoje iza biohemijskih promena masnog tkiva izazvanih rosiglitazonom.

**Metode:**

Ekspanzija ćelija je vršena u medijumu za adipocitnu kulturu dopunjenom sa 5 µg/mL insulina nakon indukcije od 2 dana. Nakon što su te ćelije tretirane rosiglitazonom 0, 0,13 mol/L i 10 µmol/L rosiglitazona tokom 48 sati, osmog dana ćelije su sakupljene i čuvane na -80 °C. Zatim su ćelije korišćene za procenu antioksidativnih enzimskih aktivnosti, nivoa mine rala i elemenata u tragovima i sastava masnih kiselina.

**Rezultati:**

Glukoza-6-fosfat dehidrogenaza i glutation reduktaza su značajno smanjene u grupama tretiranim rosiglitazonom u poređenju sa kontrolom grupom. Na, Mg, K, Ca, Cr, Fe, Ni, Cu, Zn, Rb, Sr, Cs, Ba i Pb su određeni u ćelijskim lizatima preko ICP-MS. Takođe, relativni sadržaj FAME se smanjio u grupama tretiranim rosiglitazonom u poređenju sa kontrolom grupom.

**Zaključak:**

Tretman rosiglitazonom u malim dozama pokazao je obećavajuće rezultate koji mogu pospešiti stvaranje mrkog masnog tkiva.

## Introduction

Adipose tissue stores and/or releases fatty acids (FAs) depends on the need of the energy requirement of organisms since adipocytes are the main regulators of energy and glucose homeostasis [1] [2]. White adipose tissue (WAT) and brown adipose tissue (BAT) are types of adipose tissue and coexist in mammals. WAT is responsible for energy storage in the form of triacylglycerol where BAT burns triglycerides to generate energy called as thermogenesis and decrease significantly by maturation in human. Thus, impairment in the adipose tissue function causes several diseases, including obesity, type II diabetes mellitus (T2DM), cardiovascular diseases and cancer [3] [4].

Rosiglitazone (RSG) is one of the drugs used for patients with type 2 diabetes to reduce insulin resistance and hyperglycemia via decreasing blood glucose levels. Also, this drug induces adipose differentiation, triglyceride storage (TG) and lipogenesis [5] [6] [7]. Moreover, RSG induces transdifferentiation of WAT into BAT via increasing mitochondrial mass and lipid oxidation. In this concept, developing drugs which enhance BAT formation or conversion of WAT into BAT is considered as an important strategy to fight against obesity and T2DM [1] [2] [3] [4] [8] [9].

T2DM, metabolic syndrome and obesity are characterized by the insulin resistance and the impairment in the insulin secretion which both are tightly associated with the impairment in the adipose tissue functions, and increased levels of reactive oxygen species (ROS) termed as oxidative stress [10] [11] [12] [13] [14] [15] [16] [17] [18]. Oxidative environment induces ROS to attack lipids leading to both lipid peroxidation and lipotoxicity that cause adipocyte dysfunction. Impairment in the adipocyte homeostasis results in the excessive release of free fatty acid (FFA) which are transported to the distant tissues, including liver, muscle or pancreatic cells and cause toxic effects in the target organs. Therefore, targeting adipose tissue can be an effective method to prevent diabetes, T2DM and metabolic syndrome [8] [19] [20]. Development of such therapeutics requires significant knowledge of the molecular mechanisms controlling the formation and function of brown fat cells [7]. Therefore, in this study, we aimed to investigate fatty acid composition, oxidative stress metabolism and their relation to the trace and mineral levels to reveal molecular mechanisms which control BAT into WAT conversion.

## Materials and Methods

### Materials

Protease inhibitor cocktail was obtained from Sigma-Aldrich (Germany). Glucose-6-phosphate (G6P), sodium phosphate monobasic and dibasic, 6-phosphogluconate (6-PG), reduced nicotinamide adenine dinucleotide phosphate (NADPH + H^+^), oxidized glutathione (GSSG), glutathione reductase (GR), reduced glutathione (GSH), sodium azide, hydrogen peroxide (H_2_O_2_) and ethylenediaminetetraacetic acid (EDTA), nicotinamide adenine dinucleotide phosphate (NADP^+^), magnesium chloride (MgCl_2_), Tris [Tris (hydroxymethyl) aminomethane] were purchased from Sigma-Aldrich (USA). Chemicals and organic solvents used for lipid analysis were obtained as HPLC grade. Hexane, 65% HNO_3_ of SUPRAPUR® grade, methanol and HCl were purchased from Merck (Germany). C4-C24 FAME mix standard solution and methyl tricosanoate were obtained from Sigma-Aldrich (USA) and used for FAME quantification, identification and as internal standard, respectively.

### Methods

### Preparation of primary white adipocyte culture

30-35 days-old male mice were used to obtain inguinal stroma-vascular (SV) fractions. SV cells were grown, and adipocyte differentiation was performed as described by Serkan et al. [21]. Cells were expanded in the adipocyte culture medium supplemented with 5 µg/mL insulin following two days induction. Starting at day 6, cells were grown in adipocyte culture medium in the following conditions 0, 0.1 3 mol/L and 10 µmol/L rosiglitazone for 48 hours, and at day 8, cells were collected and stored at -80 °C until experiments.

### Preparation of cell lysates

Cells were scraped off in 1 mL PBS and then transferred into 1 mL centrifuge tubes. Samples were centrifuged at 2000 rpm for 5 minutes, and then cell pellets were suspended in the 200 mmol/L sodium phosphate buffer (pH 7.4) containing protease inhibitor cocktail and sonicated for 10 seconds on the ice. Afterwards, samples were centrifuged at 14,500 rpm for 25 minutes at 4 °C and stored at -80 °C freezer until experiments.

### Glucose-6-phosphate dehydrogenase (G6PD) activity

The assay mixture was prepared with 10 mmol/L MgCl_2_, 0.6 mmol/L G6P and 0.2 mmol/L NADP^+^ in 100 mmol/L Tris/HCl buffer (pH 8.0). G6PD activity was evaluated spectrophotometrically by following NADPH production at 340 nm and 37 °C [22]. The required amount of G6PD enzyme to reduce one mmol NADP^+^/min under the indicated assay conditions was used to define one unit (U) of activity. Specific G6PD enzyme activity was represented as the number of units/mg protein.

### 6-phosphogluconate dehydrogenase (6-PGD) activity 6-Phosphogluconate dehydrogenase activity

6-Phosphogluconate dehydrogenase activity (G6PD) was determined by the same method as described above for the G6PD. Only, 0.6 mmol/L 6G6P was substituted for 0.6 mmol/L 6-PG as a substrate in the assay mixture (23).6-Phosphogluconate dehydrogenase activity (G6PD) was determined by the same method as described above for the G6PD. Only, 0.6 mmol/L 6G6P was substituted for 0.6 mmol/L 6-PG as a substrate in the assay mixture [23].

### Glutathione reductase (GR) activity

Modified Staal method was used to evaluate GR activity at 37 °C spectrophotometrically. The incubation mixture was prepared with 200 mmol/L sodium phosphate buffer (pH 7.4), 1 mmol/L GSSG, 0.2 mmol/L NADPH and cell lysate as enzyme source. The decrease in the absorbance at 340 nm was monitored for 60 seconds [24].

### Glutathione-S-transferase (GST) activity

Glutathione-S-transferase enzyme activity was determined by measuring the conjugation of GSH with 1-chloro-2, 4-dinitrobenzene (CDNB) as described by Habig et al. [25]. The activity was followed for 30 seconds at 37 °C.

### Glutathione peroxidase (GPx) activity

Incubation mixture contained 200 mmol/L potassium phosphate buffer (pH 7.4), GR enzyme (10 U/mL), 400 mmol/L sodium azide, 200 mmol/L EDTA, 100 mmol/L GSH, 2 mmol/L NADPH, 5 *μ*L tissue lysate and completed to 500 μL with double distilled water [26]. Samples were incubated for 10 min at 37 °C, and then the reaction was initiated by the addition of 5 μL of 10 mmol/L H_2_O_2_. Afterwards, GPx activity was measured spectrophotometrically for 30 seconds at the room temperature.

### Protein determination

BCA method was used to determine protein concentrations of the samples by using Spectramax M2 microplate reader. The assay was performed with the Pierce™ BCA Protein Assay Kit by following instructions [27].

### ICP-MS Analysis

Cell lysates were diluted by a factor of 10 in 65% HNO 3 of SUPRAPUR® grade. Trace and mineral element levels were determined by using Agilent 7700x ICP-MS (Agilent Technologies Inc., Tokyo, Japan) as described by Aydemir et al. [28].

### Transesterification

In situ acidic direct transesterification (DT) was used for derivatization of fatty acids according to Gomez et al. [29]. Briefly, samples were treated with 3 ml methanol with 2% HCl for 2 h at 80°C. After samples had reached the room temperature, hexane and distilled water were added to the reaction mixture to separate the fatty acid methyl esters (FAMEs).

### Fatty acids determination by gas chromatography-mass spectrometry (GC-MS)

Fatty acid methyl esters were analyzed by 7890-B Agilent GC equipped with a 5977A Agilent MSD detector and a DB-23 column (60 m × 0.25 mm × 0.25 mm film thickness). 1 mL FAME extract was used from each sample. The splitless mode was applied, and the injector temperature was set up to 250 °C. Helium was used as the carrier gas at 50 °C and 180 kPa (33 cm/S). The column temperature was increased from 50 °C to 175°C at 25 °C per min and then from 175 °C to 235 °C at 4 °C per min, and the detector temperature was 250 °C.

### Statistical Analysis

All data were analyzed by GraphPad Prism, and one-way analysis of variance (ANOVA) followed by a Tukey's post hoc test for multiple comparisons for comparison data sets. All data were represented as the mean ± standard deviation (SD).

## Results

### Anti-oxidant enzyme activities

**Figure 1:**
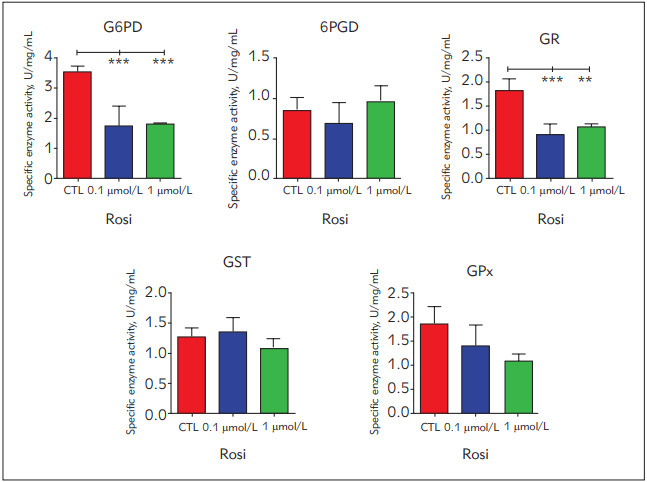
Anti-oxidant enzyme activities in control and rosiglitazone treated cells All results were given as mean ± SD. a different from control (p = 0.0004), b different from control (p = 0.0005), c different from control (p = 0.0003), d different from control (p = 0.001).

G6PD, GR and GPx activities decreased in the RSG treated cells compared to the control, however, this decrease was not significant for GPx activity. On the other hand, there was not a substantial change in the GST and 6-PGD activities (Figure 1, Table 1).

**Table 1:** Anti-oxidant enzyme activities in control and rosiglitazone treated cells All results were given as mean ± SD. ^a ^different from control (p = 0.0004), ^b ^different from control (p = 0.0005), ^c ^different from control (p = 0.0003), ^d ^different from control (p = 0.001)

Enzyme	Control	Rosiglitazone	
		0.1 µmol/L	1 µmol/L
G6PD, U/mg protein	3.53 ± 0.2	1.742 ± 0.6b^a^	1.816 ± 0.04^b^
6-PGD, U/mg protein	0.84 ± 0.17	0.68 ± 0.25	0.96 ± 0.19
GST, U/mg protein	1.8 ± 0.2	0.9 ± 0.2	1.05 ± 0.06
GR, U/mg protein	1.25 ± 0.15	1.33 ± 0.23^c^	1.05 ± 0.18^d^
GPx, U/mg protein	1.83 ± 0.3	1.37 ± 0.4	1.05 ± 0.16

### Trace element and mineral levels

**Figure 2:**
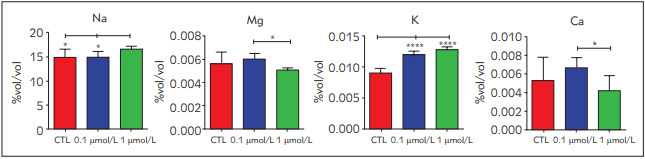
Mineral levels in control and rosiglitazone-treated groups All results were given as mean ± SD. *p < 0.05, **p < 0.01 and *** p < 0.001.

Na, Mg, K, Ca, Cr, Fe, Ni, Cu, Zn, Rb, Sr, Cs, Ba and Pb were determined in the cell lysates via ICP-MS. Ca and Mg levels significantly decreased in the 0.1 µmol/L RSG treated groups compared to the 1 µmol/L treated ones. Also, K concentrations increased in the RSG-treated groups compared to the control, where Na levels significantly decreased in control and 0.1 µmol/L RSG treated groups compared to the 1 µmol/L treatment groups (Figure 2).

**Figure 3:**
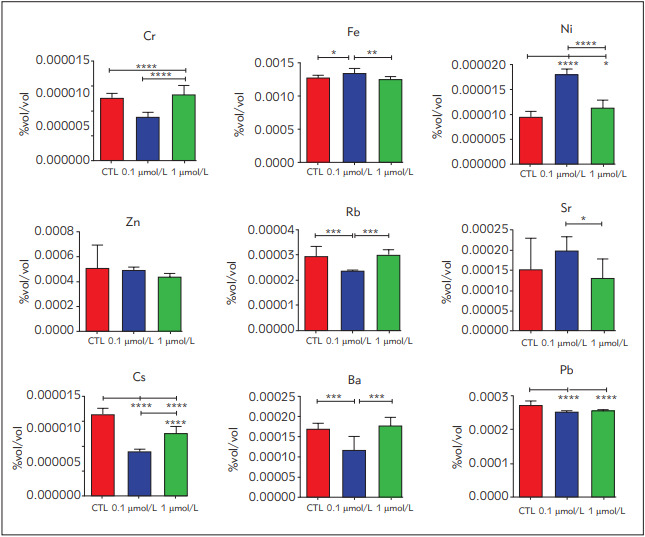
Trace element levels in control and rosiglitazone-treated groups All results were given as mean ± SD. *p < 0.05, **p < 0.01 and *** p < 0.001

On the other hand, Ba and Rb levels significantly decreased in 0.1 µmol/L dosage groups compared to the other groups, where Fe levels elevated considerably. Pb, Cs and Zn concentrations decreased in the RSG treated groups in comparison with the control. Sr and Ni levels had their peaks in 0.1 µmol/L RSG treated groups, where Cs had peak concentration in 1 µmol/L RSG dosage groups (Figure 3).

### FAME analysis

**Figure 4:**
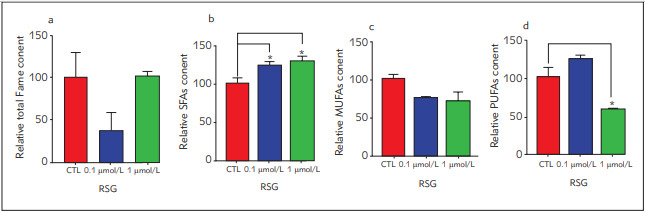
The relative amount of total fatty acid content, saturated fatty acids (SFAs), monosaturated fatty acids (MUFAs) and polyunsaturated fatty acids (PUFAs) of cells treated with 0.1 mmol/L and 1 mmol/L of rosiglitazone Total peak areas and FAME composition (% of total FAME) of samples were measured. First, the fold change was calculated between control and rosiglitazone-treated samples, and then the percentage change for each was calculated after setting the amount in control as 100%. All results were given as mean ± SD. *p < 0.05 and **p < 0.01.

**Figure 5:**
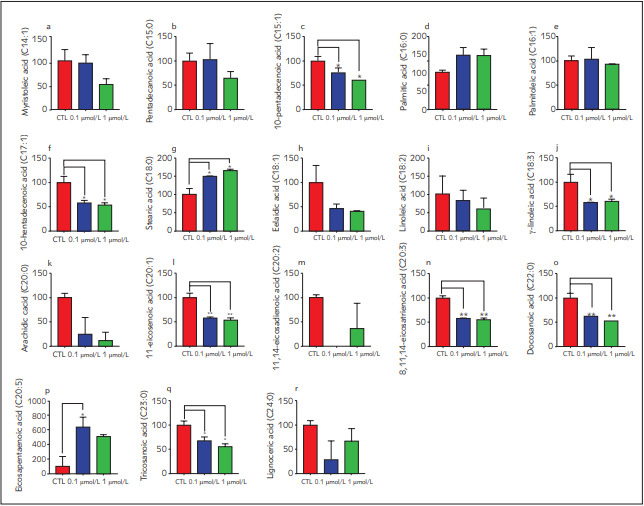
Relative amount individual and total fatty acid content of cells treated with 0.1 mmol/L and 1 mmol/L of rosiglitazone Total peak areas and FAME composition (% of total FAME) of samples were measured. First, the fold change was calculated between control and rosiglitazone-treated samples, and then the percentage change for each was calculated after setting the amount in control as 100%. All results were given as mean ± SD. *p < 0.05 and **p < 0.01

Relative FAME content decreased in RSG-treated groups compared to the control (Figure 4a) and relative saturated fatty acid (SFAs) content significantly increased in RSG treated groups compared to the control (Figure 4 b-d). Myristoleic acid, 10-pentadecenoic acid, linoleic acid, elaidic acid, 10-heptadecenoic acid, γ-linoleic acid, arachidic acid, 11-eicosenoic acid, pentadecanoic acid, palmitic acid, palmitoleic acid, stearic acid, 8, 11, 14-eicosatrienoic acid, docosanoic acid, eicosapentaenoic acid, tricosanoic acid and lignoceric acid were detected in the control and RSG-treated groups (Figure 5). All detected fatty acids decreased in RSG dosage groups compared to the control, except palmitic, stearic and eicosapentaenoic acid. However, all changes were not significant (Figure 5 a-r). On the other hand, most abundant fatty acids were palmitic and stearic acid in all groups (Figure 5).

## Discussion

Metabolic disorders, T2DM and obesity are characterized by the impairment in the insulin secretion, sensitivity and adipose tissue function. Moreover, both oxidative stress and lipid metabolism play a significant role in the pathogenesis of these diseases and targeting fat tissue can be considered as a good strategy to treat these diseases. Rosiglitazone is used to treat T2DM via reducing insulin resistance and hyperglycemia, also inducing the conversion of WAT into BAT [2] [7] [12] [16] [20]. Revealing the molecular mechanism behind the transdifferentiation of WAT into BAT is required to fight against diabetes and obesity. Therefore, in our study, we investigated the antioxidant enzyme activity, mineral and trace element levels and fatty acid composition in RSG-treated adipocytes.

G6PD, GR and GPx activities decreased in RSGtreated groups compared to the control (Figure 1, Table 1). G6PD is the rate-limiting enzyme of the pentose phosphate pathway (PPP) and responsible for cellular NADPH production involving in the fatty acid analysis. Also, G6PD contributes to the maintenance of the redox status of the cells via production of the NADPH contributing to the GR activity which converts oxidized glutathione (GSSG) into reduced glutathione (GSH). On the other hand, GPx involves in the formation of the GSSG by using GSH. Therefore, according to our data, RSG treatment reduces G6PD activity, NADPH synthesis, GR and GPx activities [30] [31].

Afterwards, we evaluated mineral and trace element levels in control and RSG-treated samples. Our data showed that Ca^+2^ levels significantly increased in the 0.1 µmol/L RSG treatment compared to the 1 µmol/L group (Figure 2). Elevated calcium levels are known to induce thermogenesis, and 0.1 µmol/L RSG treatment may trigger thermogenesis in adipocytes via β-AR signalling [32]. ATP-sensitive potassium (K_ATP_) channels play a vital role in the insulin secretion via depolarization of the cell membrane that leads to the Ca^+2^ influx in the cell, causing insulin secretion. Inhibition of ATP-sensitive potassium (K_ATP_) channels by RSG was reported before [33]. Our data showed that 0.1 µmol/L RSG treatment induced increased Ca and K levels in the cells that may show inducing the insulin secretion (Figure 2). On the other hand, Mg is essential for glycolysis, citric acid cycle, increasing insulin sensitivity and improving lipid profile [34] [35] [36] [37] [38]. We showed that 0.1 µmol/L RSG treatment caused an increase in magnesium concentrations that may be explained by the necessity of Mg in the cells to improve insulin sensitivity and the lipid profile (Figure 2).

Concentrations of various trace elements including cadmium, chromium, iron, nickel, silver, caesium, lead, rubidium and zinc were investigated in patients with T2DM. Increased Cr and Zn levels were reported in T2DM patients [39] [40]. Also, we showed decreased Cr and Zn levels in the cells upon 0.1 µmol/L RSG treatment (Figure 3). Furthermore, increased levels of heavy metals such as arsenic, zinc and lead are known by their contribution to diabetes via acting like endocrine-disrupting chemicals [39] [41]. We showed that Pb levels significantly decreased in RSG treated groups compared to the control, which can be considered as a positive response against T2DM risk upon RSG treatment (Figure 3). Li et al. reported that Ni has protective effects against diabetes and increased Cs and Ba levels were correlated with the risk of diabetes [41]. We showed that RSG treatment increased cellular Ni levels and decreased Cs and Ba levels that compromise previous data.

Furthermore, we investigated total FAME and fatty acid composition in RSG treated and control groups. Our data have revealed that 0.1 µmol/L RSG treatment reduced relative FAME content, where 1 µmol/L treatment did not show any change in the FAME content (Figure 4a). We showed that relative eicosapentaenoic acid content significantly increased in 0.1 µmol/L RSG treated group compared to the control (Figure 5p). Eicosapentaenoic acid is a PUFA which enhances insulin sensitivity and thermogenesis [42]. On the other hand, 18-carbon fatty acids including stearic and oleic acid were reported as metabolic modifiers via *in vivo* studies since they increase the thermogenic capacity of WAT [43] [44]. The relative content of both stearic acid and oleic acid increased in RSG-treated cells compared to the control according to our data that may show the contribution of RSG to the conversion of WAT into BAT (Figure 5).

According to our data, tricosanoic acid, 11eicosenoic acid, 11,14-eicosadienoic acid, docosanoic acid, 10-heptadecenoic, 10-pentadecenoic acid and γ-linolenic acid significantly decreased upon RSG treatment compared to the control (Figure 5). Since there is no publishing data related to indicated fatty acid concerning oxidative stress and mineral and trace elements, their impact on brown fat tissue and thermogenesis should be further investigated.

Overall, RSG treatment reduced G6PD and GR enzymes contributing to the oxidative stress and fatty acid metabolism. Also, RSG treatment improves trace element and mineral levels that enable restoring of insulin resistance and BAT formation. Relative FAME content reduced upon RSG treatment and relative content of some fatty acids which promote BAT formation increased. Thus the influence of fatty acid composition and types on the BAT formation should be further investigated.

## Conclusion

Adipose tissue is considered as a target for the treatment of insulin resistance and diabetes. RSG is used for the treatment of T2DM and induces transd-ifferentiation of WAT into BAT via increasing mitochondrial mass and lipid oxidation. Therefore, the development of therapeutics targeting adipocytes requires information about the molecular mechanisms controlling the formation and function of brown fat cells. RSG treatment on the adipocytes at low doses showed promising results which may address conversion of WAT into BAT. However, the impact of fatty acids on the brown fat formation should be further investigated.

*Acknowledgement*. We thank Dr Serkan KIR for primary mouse adipocytes. The authors gratefully acknowledge the use of the services and facilities of the Koc University Research Center for Translational Medicine (KUTTAM) and Koc University Tupras Energy Center (KUTEM).

## Conflict of interest statement

The authors state that they have no conflicts of interest regarding the publication of this article.

## 
